# On Defining Expressions for Entropy and Cross-Entropy: The Entropic Transreals and Their Fracterm Calculus

**DOI:** 10.3390/e27010031

**Published:** 2025-01-02

**Authors:** Jan A. Bergstra, John V. Tucker

**Affiliations:** 1 Informatics Institute, University of Amsterdam, Science Park 900, 1098 XH Amsterdam, The Netherlands; 2 Department of Computer Science, Bay Campus, Fabian Way, Swansea University, Swansea SA1 8EN, UK; j.v.tucker@swan.ac.uk

**Keywords:** partial formulae, fracterm calculus, transreals, entropic transreals, peripheral numbers, entropy, cross-entropy

## Abstract

Classic formulae for entropy and cross-entropy contain operations $\frac{x}{0}$ and $\log_{2}x$ that are not defined on all inputs. This can lead to calculations with problematic subexpressions such as $0\log_{2}0$ and uncertainties in large scale calculations; partiality also introduces complications in logical analysis. Instead of adding conventions or splitting formulae into cases, we create a new algebra of real numbers with two symbols ±∞ for signed infinite values and a symbol named ⊥ for the undefined. In this resulting arithmetic, entropy, cross-entropy, Kullback–Leibler divergence, and Shannon divergence can be expressed without concerning any further conventions. The algebra may form a basis for probability theory more generally.

## 1. Introduction

Consider a probability function, or more precisely, a probability mass function, *P* on a finite sample space *S* for which it is assumed that

$$\forall_{v\in S}P\left(v\right)\geq 0\;\mathrm{and}\;\sum_{v\in S}P\left(v\right)=1.$$

The definition of entropy for *P* is often formulated as follows:

$$H\left(P\right)=-\sum_{s\in S}\left(P\left(s\right)\cdot \log_{2}P\left(s\right)\right).$$

Alternately, it can be formulated as

$$H\left(P\right)=\sum_{s\in S}\left(P\left(s\right)\cdot \log_{2}\frac{1}{P(s)}\right).$$

A closely related concept is cross-entropy defined for two probability mass functions, say *P* and *Q*, by

$$H\left(P,Q\right)=-\sum_{s\in S}\left(P\left(s\right)\cdot \log_{2}Q\left(s\right)\right).$$

Alternately, it can be formulated as

$$H\left(P,Q\right)=\sum_{s\in S}\left(P\left(s\right)\cdot \log_{2}\frac{1}{Q(s)}\right).$$

In the formulae, there are partial functions, i.e., functions that are not defined for all the values required: neither $\log_{2}\left(x\right)$ nor $\frac{1}{x}$ are defined for x=0. However, a mass function with P(s)=0 is a valid argument in both formulae.

Correct mathematical writing practice typically guards against partiality by expressing conditions that rule out arguments or break up formulae into different cases. Another technique, one which aims to preserve uniformity, is to invent conventions for applying the formulae, which may or may not have plausible justifications. Consider the first formula. Commonly, an additional convention often prescribed is that

$$0\cdot \log_{2}0=0.$$

This has an underlying argument that

$$\lim_{x↓0}x\cdot \left(\log_{2}x\right)=0$$

to which we will return to shortly.

The convention to adopt $0\cdot \log_{2}0=0$ may seem unproblematic [1]. But, from an algebraic and logical perspective in pure mathematics, and especially from the precisely formalised logical perspectives of computer science and software engineering, introducing a convention that allows one to calculate *as if* $0\cdot \log_{2}0=0$ is not at all straightforward as it raises questions about the effects such an identification. There are two issues in need of attention: first,

(i) *What is the scope of the assumption $0\cdot \log_{2}0=0$? Is it only local to the definitions of entropy and cross-entropy or is the scope more extensive.*

If the assumption is made more generally, for calculations in theoretical work on entropy, one may wonder about the value of other expressions, such as $0\cdot \log_{2}^{2}0,0\cdot \log_{2}^{3}0$, etc.

(ii) *If $0\cdot \log_{2}0=0$, then what is the status of the log term $\log_{2}0$?*

The question applies to the equally relevant term $\frac{1}{0}$ just as well, of course. Now, these formulae have long been very widely used in computing; however, of theoretical interest for programming is the question:

(iii) *What are the effects of the inherent partiality of the formulae being disguised?*

Partial operators on inputs that sometimes return no output are to be avoided in programming, and logical reasoning about programs becomes hugely more complicated than with total operators.

In this paper, we will adopt a foundational approach and explore the partiality of these entropy formulae—and of several derived information-theoretic formulae—in order to develop some new algebraic structures for real number arithmetic that can provide single uniform expressions that are well founded because they are based not on ad hoc conventions but on the algebra of the number systems that underpin the formulae.

To accomplish this, we will need to examine technique(s) for making partial functions total and detecting their effect on calculations. In pursuing one technique, that of using infinities, we develop an arithmetic algebra of real numbers customised to our problem and hence called *entropic transreals*. In the entropic transreals, the convention $0\cdot \log_{2}0=0$ becomes an authentic algebraic property, derivable from the algebra of entropic transreals.

### 1.1. Gauging the Problem

In addition to addition, subtraction and multiplication (which are total operations), the algebra of real numbers we aim to construct will also have division $\frac{x}{y}$ and $\log_{2}x$ (which are partial operations that will need to be made total). What factors shape the design of the new algebra? For example, the ubiquitous presence of summation over a sample space, as in the definitions of entropy, suggests the requirement that addition is associative.

Consider how the convention $0\cdot \log_{2}0=0$ could be a valid equation in such an algebra. If one relies on an justification of the value of $0\cdot \log_{2}0$ by way of limits, as mentioned earlier, then applying the multiplication rule for limits we deduce

$$0=\lim_{x↓0}\left(x\cdot \left(\log_{2}x\right)\right)=\lim_{x↓0}\left(x\right)\cdot \lim_{x↓0}\left(\log_{2}x\right)=0\cdot -\infty .$$

Thus, we have another rather basic identity, 0=0·−∞, to consider and the idea that $\log_{2}0=-\infty$.

Turning to cross-entropy, in some cases one expects it to take a positive infinite +∞ value, and so an arithmetic equipped with both signed infinities ±∞ is needed.

**Example 1.**  *Consider a sample space S with elements a and b and probability mass functions P and Q over S such that*

P(a)=1,P(b)=0,Q(a)=0,Q(b)=1.

*Now, for the cross-entropy H(P,Q), as it was defined above, we expect to find value +∞. Calculation of H(P,Q) yields:*

$$H\left(P,Q\right)=P\left(a\right)\cdot \log_{2}\frac{1}{Q(a)}+P\left(b\right)\cdot \log_{2}\frac{1}{Q(b)}=1\cdot \log_{2}\frac{1}{0}+1\cdot \log_{2}\frac{1}{1}=\log_{2}\frac{1}{0}.$$

So, to obtain the expected value +∞ in this case, we can adopt the identity $\frac{1}{0}=+\infty$, in combination with $\log_{2}\left(+\infty \right)=+\infty$; this seems plausible, if not necessary.

Thus, with these and other requirements in mind, in the course of the paper, the field of real numbers will be enriched with new operations and elements to make a new algebra. In particular, we will extend it with a suitable pair of signed infinite ‘values’ ±∞—elements outside the conventional range of numbers. On adopting $\log_{2}0=-\infty$ and enabling 0·∞=0·(−∞)=0 to hold, the desired convention $0\cdot \log_{2}0$ can derive an algebraic property of the underlying arithmetical data type.

### 1.2. Structure of the Paper

In Section 2, we prepare the ground with some background and methods for treating partiality that have been developed for division. In Section 3, we continue to explore and select algebraic properties customised to the task of rebuilding the entropic formulae. In Section 4, we apply the new entropic transreals to a series of formulae for entropy, cross-entropy, Kullback–Leibler divergence, and Shannon divergence. In Section 5, we summarise the construction of the algebra. In Section 6, we reflect on the exercise and point out some next steps and problems.

## 2. Peripheral Numbers, Fracterms, Fracterm Calculus

Technically, we are concerned with the use of expressions $\frac{1}{0}$ and $\log_{2}0$ that have no values and finding ways to give them meaning for the purpose of improving calculating and reasoning. The tools we employ are made from a variety of logical concepts and methods concerning equations and related formulae that make up the theory of abstract data types in computer science [2]. However, to keep focussed on entropy, we will limit the use of this background knowledge that informs our investigation.

### 2.1. Peripherals

Our methods benefit from an elementary knowledge of syntax, namely *signatures*, which list names for constants and operators of an algebra, and the *terms* that are made by composing operators and applying them to constants and variables.

The constants ⊥ and ∞ are new syntax, from a conventional point of view, though ∞ is used quite often in an informal manner, and these constants at the same time represent values outside the conventional number system, so-called *peripheral numbers*. We will consider arithmetical structures, or arithmetics for short, which feature three peripheral values: ∞, −∞ and ⊥. We will sometimes write +∞ for ∞ to emphasize that positive infinity is meant.

In introducing new infinity constants ∞, −∞ we generate the need for meaning for infinitely many new expressions:

$$\infty +\infty ,\;\infty \cdot \infty ,\;\infty -\infty ,\;\frac{\infty }{\infty },\;\ldots ,\;\log_{2}+\infty ,\;\log_{2}-\infty ,\;\ldots$$

Some seem easy to resolve with identities, such as

∞+∞=∞,∞·∞=∞,

while others suggest options, such as

$$\infty -\infty =?,\;\frac{\infty }{\infty }=?,$$

and the choices and the algebras they determine ramify. In our case, we will use ⊥ in identities to resolve the matter.

Following [3], we use the word *fracterm* for a fractional expression. We avoid the noun “fraction” because its meaning is rather ambiguous, ranging between an expression and its number value. Whereas for constants making a distinction between expression and value is rather uninformative, for fractional expressions it matters a lot. We adopt $\frac{1}{0}$ as a fracterm without hesitation. We use *fracterm calculus* loosely for “how to calculate with fracterms”. Different fracterm cacluli may be distinguished and axiomatised using formulae based on equations, for instance.

### 2.2. Models for Division by Zero

In this paper, we start from a series of thorough studies of the case of division: what can be done about $\frac{x}{0}$? There are several options that have been analysed.

(i) *Suppes-Ono fracterm calculus.* This is based on the assumption $\frac{x}{0}=0$ and makes no use of ⊥. For information on Suppes-Ono fracterm calculus, we refer to [4,5,6,7,8].

(ii) *Common meadows fracterm calculus.* This uses $\frac{x}{0}=⊥$ and makes no use of ∞ and −∞. We refer to [9,10,11] for common meadows.

(iii) *Transreal fracterm calculus.* This uses Φ (instead of ⊥ and named nullity), ∞ and −∞. See [12,13,14,15,16,17].

(iv) *Fracterm calculus for symmetric transreals.* This involves peripherals for signed infinitesimals, as well as for signed infinities. See [18].

(v) *Fracterm calculus for wheels.* This makes use of ⊥ while identifying ∞ and −∞ and maintaining ∞+∞=⊥. See [19].

Below, we will propose an adaptation of transreal fracterm calculus by introducing ⊥ besides Φ and adopting $\log_{2}p=⊥$ rather than $\log_{2}p=\Phi$ for negative *p*, in order to find a better alignment between different fracterm calculi. This leads us to our *Fracterm calculus for entropic transreals.*

Entropic transreals are an approach to enlargement of arithmetic with peripheral numbers (which will be introduced below) that is designed specifically to meet the objective to provide a precise meaning for defining expressions for entropy and cross-entropy. We will speak of *entropic* transreals—to emphasise their motivation. Arguably, the entropic transreals embody somewhat arbitrary assumptions, e.g., the combination of 0·∞=0 and ∞−∞=⊥ may be called rather ad hoc.

### 2.3. Indicating Partiality

We will adopt these conventions: instead of “$f(a_{1},\ldots ,a_{n})$ is undefined” we write $f(a_{1},\ldots ,a_{n})=⊥$. Thus, ⊥ is considered an element of the domain of values. So ⊥ plays a conventional role as an element of the domain in the setting of equational logic with the effect that, for instance, ⊥=⊥ and ⊥≠0. We often refer to ⊥ as an “error value”, but its role as a token for partiality need not be understood as signalling an error.

We assume that $\log_{2}$ is undefined for negative arguments, though following the approach of [9] and adopting the mechanism of quasi-partiality we prefer to work with total functions writing f(a)=⊥ in case one thinks of f(−) as being undefined for argument *a*, a consideration which leads to $\log_{2}p=⊥$ for any real number p<0, as well as to $\log_{2}\left(-\infty \right)=⊥$.

When making use of a square root function $\sqrt{x}$ we will adopt $\sqrt{p}=⊥$ for negative real *p* as well as $\sqrt{-\infty }=⊥$. Adopting f(a)=⊥ only indicates that no proper value is assigned to f(a) in the setting at hand, while it may be the case that in a larger structure (such as a field of complex numbers) such values may be easily found. With the use of ⊥ in these cases we deviate from transreals (as in [12]) where $\log_{2}\left(-1\right)=\Phi$ is assumed.

## 3. Entropic Transreals

In various floating point systems for computer arithmetic one finds $\frac{1}{0}=+\infty$ and $\frac{-1}{0}=-\infty$. Remarkably, however, within theoretical computer science these ubiquitous conventions have not lead to any systematic research on versions of arithmetic with peripheral numbers for signed infinities. We are unaware of any occurrence of ∞ with the properties of entropic transreals (perhaps with another name or symbol) in the literature. The design and elaboration of transreal arithmetic stands out as a rather singular example.

### 3.1. The Fracterm Calculus of Transreals Fails to Match Our Requirements

The best-known instance of a version of arithmetic providing peripheral numbers ±∞ is the system of transreals as defined in [13]. Transreals contain an absorptive constant Φ, named nullity, that satisfies

0·∞=0·(−∞)=x+Φ=x·(−∞)=Φ.

It follows that, upon adopting $\log_{2}0=-\infty$, one obtains for the convention

$$0\cdot \log_{2}0=0\cdot \Phi =\Phi$$

instead of the desired $0\cdot \log_{2}0=0$. Thus, transreal arithmetic will not support the definition of entropy in its conventional form.

Moreover, for any probability mass function *P* on *S* which vanishes on at least one sample s∈S, one finds H(P)=Φ when adopting the conventional definition of entropy in combination with the conventions of transreal arithmetic.

Transreal arithmetic was designed with the IEEE 754 standard in mind (see also [14]), and maintaining definitions from probability theory without any modification has been no requirement on the design of the fracterm calculus of transreals.

### 3.2. The Fracterm Calculus of Entropic Transreals

We will adopt entropic transreals, an enlargement of reals with different periperhal numbers ±∞ such that

0·∞=0·(−∞)=0.

The simplification with respect to transreals lies in the fact that the familiar identity 0·x=0 is maintained to a greater extent. In other words, the role of nullity (Φ) is reduced, and in fact so much reduced that its remaining role is played by the partiality indicator ⊥. Notice that with the error element ⊥, we will be using 0·⊥=⊥ rather than 0·⊥=0, so that the familiar equation 0·x=0 is again compromised in entropic transreals, though to a lesser extent than in transreals where 0·∞=0·(−∞)=Φ is adopted.

With ±∞ available, we will follow the design of transreals as in [12] and adopt the following equations: $\log_{2}0=-\infty$ and $\log_{2}\infty =\infty$. Finding a value for $\log_{2}-1$ and for $\log_{2}-\infty$ is another matter, however.

In Section 5, we will summarise in full detail the domain, the constants, and the various operators of entropic transreals.

### 3.3. The Role of ⊥

The peripheral value ⊥ is needed for entropic transrationals in order to evaluate the sumterm ∞+(−∞). We will adopt for entropic transrationals the identity ∞+(−∞)=∞−∞=⊥, an equation which is already valid for transrationals. This assumption is consistent with the requirement that addition and multiplication are associative. We notice that both associativity and commutativity of addition are needed for generalized addition over a finite domain to have a plausible interpretation. Generalized addition occurs in the definition of entropy and of expected value in general.

Although ⊥ is not included in transreals, we consider entropic transreals with ⊥ for quasi-partiality is still to be a simplification of transreals. In transreals, Φ is not supposed to play the role of ⊥, and therefore Φ is not supposed to model partiality in general: in the design of transreals, Φ is instead the meaningful value of 0·∞. In our case, $\log_{2}\left(-3\right)$ is not supposed to have a meaningful value, so that we consider it plausible to set $\log_{2}\left(-3\right)=⊥$ in transreals, as well as in entropic transreals.

### 3.4. Dealing with Non-Distributivity

Just as with the transreals, the entropic transreals are not distributive. For transreals, assuming distributivity leads to the following inconsistency:

∞=1·∞=(1+0)·∞=1·∞+0·∞=∞+Φ=Φ.

For entropic transrationals, upon assuming distributivity, one finds an inconsistency as well:

⊥=∞+(−∞)=(1+(−1))·∞=0·∞=0.

Although failure of distributivity is unpleasant, it appears not to constitute a fundamental obstacle for the use of a particular arithmetical data type. Using conditional equations, several useful versions of distiributivity can be found, for instance: 0·x=0→x·(y+z)=x·y+x·z.

The lack of distributivity can be expressed without making use of constants for peripheral numbers (i.e., ∞ or ⊥) and it is the following well-known rule that fails for x=1, y=−1, z=0:

$$\frac{x}{z}+\frac{y}{z}=\frac{x+y}{z}.$$

## 4. Application to Entropy, Cross Entropy and Other Concepts

We consider a series of formulae and make some calculations using the entropic transreals as examples.

### 4.1. An Expression for Entropy

The second expression for entropy in the Introduction introduces an issue of division by zero:

$$H\left(P\right)=\sum_{s\in S}\left(P\left(s\right)\cdot \log_{2}\frac{1}{P(s)}\right).$$

In case for some s∈S, P(s)=0, for this expression it is plausible to follow the conventions of transreals as follows:

$$\frac{1}{0}=+\infty ,\frac{-1}{0}=-\infty ,\frac{1}{+\infty }=\frac{1}{-\infty }=0.$$

For a summand coming from a sample *s* with P(s)=0 we find:

$$P\left(s\right)\cdot \log_{2}\frac{1}{P(s)}=0\cdot \log_{2}\frac{1}{0}=0\cdot \log_{2}\infty =0\cdot \infty =0,$$

an outcome which we consider to be adequate for the definition of entropy.

We begin a series of running examples as a simple check and illustration of calculating with the formulae.

**Example 2.**  *Consider S={a,b} and $P\left(a\right)=P\left(b\right)=\frac{1}{2}$ while Q(a)=1 and Q(b)=0. We find for P and Q, in this case:*

$$H\left(P\right)=P\left(a\right)\cdot \log_{2}\frac{1}{P(a)}+P\left(b\right)\cdot \log_{2}\frac{1}{P(b)}=\frac{1}{2}\cdot \log_{2}\frac{1}{\left(\frac{1}{2}\right)}+\frac{1}{2}\cdot \log_{2}\frac{1}{\left(\frac{1}{2}\right)}=\;\frac{1}{2}\cdot \log_{2}2+\frac{1}{2}\cdot \log_{2}2=1$$

*and*

$$H\left(Q\right)=Q\left(a\right)\cdot \log_{2}\frac{1}{Q(a)}+Q\left(b\right)\cdot \log_{2}\frac{1}{Q(b)}=1\cdot \log_{2}\frac{1}{1}+0\cdot \log_{2}\frac{1}{0}=\;1\cdot 0+0\cdot \log_{2}\left(+\infty \right)=0+0\cdot \left(+\infty \right)=0.$$

Evaluating H(P) and H(Q) with the first definition of entropy will produce the same value because we are working with the native equality in an algebra on entropic transreals.

### 4.2. Cross-Entropy

Cross-entropy is defined for two probability mass functions, say *P* and *Q*, as follows:

$$H\left(P,Q\right)=\sum_{s\in S}\left(P\left(s\right)\cdot \log_{2}\frac{1}{Q(s)}\right)$$

**Example 3.**  *Again, take S={a,b} and $P\left(a\right)=P\left(b\right)=\frac{1}{2}$ while Q(a)=1 and Q(b)=0. We calculate:*

*For H(P,Q), we find:*

$$H\left(P,Q\right)=P\left(a\right)\cdot \log_{2}\frac{1}{Q(a)}+P\left(b\right)\cdot \log_{2}\frac{1}{Q(b)}=\frac{1}{2}\cdot \log_{2}\frac{1}{1}+\frac{1}{2}\cdot \log_{2}\frac{1}{0}=\;\frac{1}{2}\cdot \log_{2}1+\frac{1}{2}\cdot \log_{2}\infty =\frac{1}{2}\cdot 0+\frac{1}{2}\cdot \infty =0+\infty =\infty .$$

*For H(Q,P), we find:*

$$H\left(Q,P\right)=Q\left(a\right)\cdot \log_{2}\frac{1}{P(a)}+Q\left(b\right)\cdot \log_{2}\frac{1}{P(b)}=1\cdot \log_{2}\frac{1}{\left(\frac{1}{2}\right)}+0\cdot \log_{2}\frac{1}{\left(\frac{1}{2}\right)}=\;1\cdot \log_{2}2+0\cdot \log_{2}2=1\cdot 1+0\cdot 1=1.$$

*Notice that H(P,Q)≠H(Q,P).*

### 4.3. Alternative Expression for Cross-Entropy

The other definition of cross entropy reads:

$$H\left(P,Q\right)=-\sum_{s\in S}\left(P\left(s\right)\cdot \log_{2}Q\left(s\right)\right)$$

This definition depends on the basic assumptions of entropic transreal arithmetic, though in a different manner, now making use of $\log_{2}0=-\infty$.

**Example 4.**  *Again, take S={a,b} and $P\left(a\right)=P\left(b\right)=\frac{1}{2}$ while Q(a)=1 and Q(b)=0. We calculate:*

$$H\left(P,Q\right)=-P\left(a\right)\cdot \log_{2}Q\left(a\right)-P\left(b\right)\cdot \log_{2}Q\left(b\right)=-\frac{1}{2}\cdot \log_{2}1-\frac{1}{2}\cdot \log_{2}0=\;-\frac{1}{2}\cdot \log_{2}1-\frac{1}{2}\cdot \left(-\infty \right)=-\frac{1}{2}\cdot 0+\frac{1}{2}\cdot \infty =0+\infty =\infty ,$$

*and*

$$H\left(Q,P\right)=-Q\left(a\right)\cdot \log_{2}P\left(a\right)-Q\left(b\right)\cdot \log_{2}P\left(b\right)=-1\cdot \log_{2}\frac{1}{2}-0\cdot \log_{2}\frac{1}{2}=\;-1\cdot \log_{2}-1-0\cdot 1=1.$$

We obtain the same values.

### 4.4. A Modification of the Example

The running example above can be modified by adding a new sample element *c*:

**Example 5.**  *We consider $S^{\prime }=S\cup \left\{c\right\}=\left\{a,b,c\right\}$ and extend the definitions of P and Q with values on c. Define $P^{\prime }\left(a\right)=P^{\prime }\left(b\right)=\frac{1}{2},P^{\prime }\left(c\right)=0$ while $Q^{\prime }\left(a\right)=1$ and $Q^{\prime }\left(b\right)=Q^{\prime }\left(c\right)=0$.*

*Now, we find:*

$$H\left(P^{\prime }\right)=H\left(P\right)+P^{\prime }\left(c\right)\cdot \log_{2}\frac{1}{P^{\prime }\left(c\right)}=1+0\cdot \log_{2}\frac{1}{0}=1+0\cdot \log_{2}\infty =1+0\cdot \infty =1+0=1,$$

$$H\left(Q^{\prime }\right)=H\left(Q\right)+Q^{\prime }\left(c\right)\cdot \log_{2}\frac{1}{Q^{\prime }\left(c\right)}=0+0\cdot \log_{2}\frac{1}{0}=0\cdot \log_{2}\infty =0\cdot \infty =0.$$

*and for cross entropy*

$$H\left(P^{\prime },Q^{\prime }\right)=H\left(P,Q\right)+P^{\prime }\left(c\right)\cdot \log_{2}\frac{1}{Q^{\prime }\left(c\right)}=\infty +0=\infty .$$

### 4.5. Kullback–Leibler Divergence

Kullback–Leibler divergence is not symmetric on the above example for *P* and *Q*:

$$D_{\mathrm{KL}}\left(P||Q\right)=H\left(P,Q\right)-H\left(P\right)=\infty -1=\infty$$

and

$$D_{\mathrm{KL}}\left(Q||P\right)=H\left(Q,P\right)-H\left(Q\right)=1-0=1.$$

We find, as is well known, that already on probability mass functions that vanish nowhere, $D_{\mathrm{KL}}\left(-,-\right)$ is asymmetric.

**Example 6.**  *Here is a new probability mass function R given by $R\left(a\right)=\frac{1}{3}$ and $R\left(b\right)=\frac{2}{3}$. We find:*

$H\left(R\right)=-\frac{1}{3}\cdot \log_{2}\frac{1}{3}-\frac{2}{3}\cdot \log_{2}\frac{2}{3}=\frac{1}{3}\cdot \log_{2}3+\frac{2}{3}\cdot \log_{2}3-\frac{2}{3}\log_{2}2=\log_{2}3-\frac{2}{3}.$

*
$H\left(P,R\right)=-P\left(a\right)\cdot \log_{2}R\left(a\right)-P\left(b\right)\cdot \log_{2}R\left(b\right)=-\frac{1}{2}\cdot \log_{2}\frac{1}{3}-\frac{1}{2}\cdot \log_{2}\frac{2}{3}=\frac{1}{2}\cdot \log_{2}3+\;\frac{1}{2}\cdot \log_{2}3-\frac{1}{2}\cdot \log_{2}2=\log_{2}3-\frac{1}{2},\;\mathrm{and}\;\mathrm{so}$
*

$$D_{\mathrm{KL}}\left(P||R\right)=H\left(P,R\right)-H\left(P\right)=\log_{2}3-\frac{1}{2}-1=\log_{2}3-\frac{3}{2}.$$

*Moreover, we have: $H\left(R,P\right)=-R\left(a\right)\cdot \log_{2}P\left(a\right)-R\left(b\right)\cdot \log_{2}P\left(b\right)=-\frac{1}{3}\cdot \log_{2}\frac{1}{2}-\frac{2}{3}\cdot \;\log_{2}\frac{1}{2}=1\;\mathrm{and}\;\mathrm{so}$*

$$D_{\mathrm{KL}}\left(R||P\right)=H\left(R,P\right)-H\left(R\right)=1-\left(\log_{2}3-\frac{2}{3}\right)=\frac{5}{3}-\log_{2}3.$$

### 4.6. Mutual Information

An instance of Kullback–Leibler divergence is so-called *mutual information*, where S=U×V, and *R* is a probability mass function on *S* with marginals *P* and *Q*, i.e.,

$$P\left(u\right)=\sum_{v\in V}R\left(u,v\right)\;\mathrm{and}\;Q\left(v\right)=\sum_{u\in U}R\left(u,v\right).$$

Now,

$$I\left(R\right)=D_{\mathrm{KL}}\left(R||P\cdot Q\right)=\sum_{u\in U,v\in V}R\left(u,v\right)\cdot \log_{2}\frac{R(u,v)}{P(u)\cdot Q(v)}$$

We notice that a probability mass function cannot have values ∞, −∞ or ⊥, and, moreover, if either P(u)·Q(v)=0 then necessarily also R(u,v)=0 so that I(R) is guaranteed to be finite, i.e., have no peripheral value.

That $\frac{0}{0}=0$ is the only nontrivial property needed of the underlying arithmetic for the above definition of I(R) to be adequate.

### 4.7. Jensen–Shannon Divergence

Given probability mass functions *P* and *Q* on *S*, the *Jensen–Shannon divergence* is as follows:

$$D_{\mathrm{JS}}\left(P||Q\right)=\frac{D_{\mathrm{KL}}\left(P||M\right)+D_{\mathrm{KL}}\left(Q||M\right)}{2}$$

where $M=\frac{P+Q}{2}$. We notice that for all *P* and *Q*, $D_{\mathrm{JS}}\left(P||Q\right)\neq \infty$. Otherwise, for some s∈S, M(s)=0 must hold together with either P(s)≠0 or P(s)≠0 (or both), which is impossible given the definition of *M*.

### 4.8. Expected Value

Both entropy and cross-entropy are instances of an *expected value*, obtained upon choosing a suitable function on the sample space. Defining an expected value operator, given a probability mass function and a function from the sample space to (real) numbers, requires an additional definition, however.

For a function *F* from a finite sample space *S* to reals (or rather to entropic transreals) and a probability mass function *P*, the following definition of an expected value ${\hat{E}}_{P,F}^{S}$ is plausible:

$${\hat{E}}_{P}^{S}\left(F\right)=\sum_{x\in S,P(x)\neq 0}P\left(x\right)\cdot F\left(x\right)$$

The virtue of this form is that unlike $E_{P}^{S}\left(F\right)=\sum_{x\in S}P\left(x\right)\cdot F\left(x\right)$ it works well (i.e., avoids result ⊥) in case for some a∈S, P(a)=0 while F(a)=⊥.

However, in fact, we prefer that latter property of an expected value operator, i.e., whenever F(a)=⊥ for some a∈S, then the expected value of *F* on *S* equals ⊥, independently of the probability mass function at hand. For that reason, we will adopt

$$E_{P}^{S}\left(F\right)=\sum_{x\in S}P\left(x\right)\cdot F\left(x\right)$$

as an appropriate definition of expected value and so that $E_{P}^{S}\left(F\right)=⊥$ whenever for some a∈S, F(a)=⊥. Adopting a convention of this kind expresses the idea that an event with probability 0 is not altogether impossible, its probability is merely extremely low. We refer to [20] for an exposition on this somewhat unconventional position regarding the status of zero-probability events.

Now, we may reformulate the defining expressions for entropy, cross-entropy and Kullback–Leibler divergence as follows:

$$H\left(P\right)=E_{P}^{S}\left(\frac{1}{P(s)}\right)$$

$$H\left(P,Q\right)=E_{P}^{S}\left(\frac{1}{Q(s)}\right)$$

$$D_{\mathrm{KL}}\left(P||Q\right)=E_{P}^{S}\left(\frac{P(s)}{Q(s)}\right)$$

## 5. Entropic Transreals in Detail

We will now describe entropic transreals in minute detail in order to prevent any confusion. The starting point is a field R of reals with constants 0 an 1 and functions addition, additive inverse, and multiplication. To this field we add operators $\frac{x}{y}$ for division and and $\log_{2}x$ for logarithm. (We only discuss functions which play a role in the paper, other functions such as exponentiation and square root might be included as well.) We also assume the presence of an ordering which we handle using a sign operator s(x) defined for:

x>0, s(x)=+1,x=0, s(x)=0,x<0, s(x)=−1.

The domain R of real numbers is enlarged by extending the domain with three new elements: ∞, −∞ and ⊥. Thus, the form of the algebra is:

$$\left(R\cup \left\{+\infty ,-\infty ,⊥\right\}|\;0,1,+\infty ,-\infty ,⊥,+,-,\cdot ,÷,\log_{2},s\left(x\right)\right)$$

Let $R_{⊥,\pm \infty }=R\cup \left\{+\infty ,-\infty ,⊥\right\}$ denote the domain.

We now have to define the operations on the three peripherals.

As ⊥ is absorptive, the value of any operation on arguments at least one of which equals ⊥ is ⊥. So, we need not specify in detail values of operators in case one of the arguments is ⊥. We turn to the infinities, which can be subtle.

*Sign function.* This is easily extended to the larger domain $R_{⊥\pm \infty }$ as follows:
  s(∞)=1,s(−∞)=−1,s(⊥)=⊥.
*Addition.* This is extended as follows: for p∈R:
  ∞+p=∞ and −∞+p=−∞;
  ∞+∞=∞ and (−∞)+(−∞)=−∞;
  ∞+(−∞)=(−∞)+∞=⊥.
*Multiplication.* This is extended as follows: for p∈R,
  0·∞=0;
  p>0, p·∞=∞ and p·(−∞)=−∞;
  p<0, p·∞=−∞ and p·(−∞)=∞.
Multiplication is taken to be commutative.
*Division.* This is defined by:
  $$\frac{x}{y}=x\cdot \frac{1}{y};$$
  $$\frac{1}{0}=\infty ;$$
  $$\frac{1}{\infty }=\frac{1}{-\infty }=0.$$
*Logarithm.* This works for p∈R:
  $$p<0,\;\log_{2}\left(p\right)=⊥;$$
  $$\log_{2}\infty =\infty ;$$
  $$\log_{2}\left(-\infty \right)=⊥.$$

### Some Properties of Entropic Transreals

The algebra of entropic transreals has several properties that are worth mentioning and are easy to prove:

**Proposition 1.** 

*(i)*  *Addition and multiplication are associative and commutative,*
*(ii)*  *x+0=x,*
*(iii)*  *x·1=1,*
*(iv)*  *x≠⊥→0·x=0,*
*(v)*  *$\frac{x}{y}=x\cdot \frac{1}{y}$,*
*(vi)*  *(x≠∞∧x≠−∞)→x+(−x)=0·x,*
*(vii)*  *x+(−x)≠⊥→x+(−x)=0.*

## 6. Concluding Discussion

The question we have raised is, *Can we design algebras that enlarge the real numbers so that some information theoretic formulae do not require conventions or special conditions to guard against partiality?* Such a question is particularly relevant to computing as such algebras are needed to design data types for programming. That partiality needs to be avoided or managed is essential in programming to avoid unwanted semantic behaviour and enable formal logical and automated tools for reasoning about programs.

Starting with an algebraic structure called the transreals (Section 3.1), a modification has been proposed for our purposes that we have named the entropic transreals. We have shown that the entropic transreals allows us to provide a suitable algebra for real arithmetic so that the formuale that arise in the conventional definitions of entropy and cross-entropy for probability mass functions on finite sample spaces are well defined.

### 6.1. On Conventions and the ‘Legality’ of Texts

Following the convention that we refer to an arithmetical expression with division as its leading symbol as a fracterm, we may refer to a expression with $\log_{2}\left(-\right)$ as its leading function symbol as a *logterm*, or when more detail is needed a *logterm with base 2*. Explanations of the definition of entropy often mention the convention that the expression $0\cdot \log_{2}0$ is understood to take value 0 to complete the formula. Upon supposing $0\cdot \log_{2}0=0$, we asked what to think of the logterm $\log_{2}0$. There seems to be no principled impediment against writing expression that contain $\log_{2}0$ as a subterm, which is in remarkable contrast with the fracterm $\frac{1}{0}$. This textual point is the subject of an investigation in [21] of conventions for notions of legality, where a text about or involving elementary arithmetic is illegal if it makes use of division by zero. Although the logterm $\log_{2}0$ makes no more sense than the fracterm $\frac{1}{0}$, both seemingly meaningless expressions are treated rather differently. We have no convincing explanation for such differences.

Entropic transreals demonstrate the consistency of the assumption $0\cdot \log_{2}=0$ by assigning the value −∞ to $\log_{2}0$ and adopting $\frac{1}{-\infty }$. These assumptions hold for transreals. Providing a grounding for the definition of transreals, however, requires the assumption that 0·∞=0, which leads to a deviation from the design of transreals, leading to what we call entropic transreals.

### 6.2. Probability Theory in the Context of Entropic Transreals

Entropric transreals are of use when dealing with definitions in connection with entropy. Perhaps the scope of the entropic transreals may extended to become a point of departure for a systematic formal logical analysis of the basics of probability. To illustrate the idea, consider a precise formulation of the well-known *Bayes–Price theorem* on inverse probability, which contains a possible division by zero in the formula.

Using the fracterm calculus of entropic transreals, the equation

$$P\left(x|y\right)=\frac{P(y|x)\cdot P(x)}{P(y)}\;\left(★\right)$$

is valid under all conditions. Indeed, if P(y)=0, then P(x∧y)=0 and

$$P\left(x|y\right)=\frac{P(x\wedge y)}{P(y)}=\frac{0}{0}=0$$

and if P(y)≠0, even including the case that P(y)=⊥ then (★) follows trivially.

So, how division by zero is handled generates conditions that need to be imposed on the formula. These can depend upon the details of the fracterm calculus that is used. For instance, in Suppes-Ono arithmetic (i.e., working with $\frac{x}{0}=0$), the equation (★) can be stated without conditions such as P(x)≠0 and/or P(y)≠0. For an application of Suppes-Ono fracterm calculus to probability theory, we refer to [22]. In fact, the results of [22] can be developed almost without modification when making use of entropic transreals instead of reals with Suppes-Ono division.

### 6.3. Potential Applications

Entropic transreals may be useful when formalizing calculations and proofs involving entropy with an eye on subsequent automated proof checking. We feel that any systematic approach to logical reasoning about entropy and cross-entropy requires a level of precision concerning the status of problematic expressions beyond the nowadays customary conventions.

Outside probability theory, entropic transreals may be helpful for designing alternative approaches to floating-point arithmetic just as transreals may be helpful for that purpose. When understanding ±∞ as overflow values, the axiom/convention of entropic transreals that 0·∞=0·−∞=0 is at least as plausible as the assumption 0·∞=0·−∞=Φ of transreal arithmetic which models the currently popular floating-point standard.

In the introduction, we qualify certain conventions as used in many expositions on entropy and cross-entropy as ad hoc. Undeniably, some design decisions made for the design of entropic transreals may also be qualified as being ad hoc. The difference is, however, that the latter design decisions are made in the systematic framework of the design of abstract datatypes, i.e., algebra in the framework of universal algebra.

## Data Availability

No new data were created or analyzed in this study. Data sharing is not applicable to this article.
